# Supplementary material for: Human Exposure to Bisphenols, Parabens, and Benzophenones, and Its Relationship with the Inflammatory Response: A Systematic Review
Source: Int J Mol Sci. 2023 Apr 15;24(8):7325. doi: 10.3390/ijms24087325 (PMC10139086; doi:10.3390/ijms24087325)
Supplement: Supplementary file 1 [file ijms-24-07325-s001.zip › ijms-2288287-supplementary.pdf]

Supplementary Table S1. Search strategies for Pubmed, Scopus and Web of Science

Database	Pubmed (Medline)
**Date**	**2/1/2023**
**Strategy**	**#1 AND #2**
#1	("bisphenols" [Title/Abstract] OR "bisphenol A" [Title/Abstract] OR "bisphenol S" [Title/Abstract] OR "bisphenol F" [Title/Abstract] OR "bisphenol A-glycidyl methacrylate" [Mesh] OR "bisphenol A-glycidyl methacrylate" [Title/Abstract] OR "bisphenol A diglycidyl ether" [Title/Abstract] OR "bisphenol F diglycidyl ether" [Title/Abstract] OR "parabens" [Mesh] OR "parabens" [Title/Abstract] OR "methylparaben" [Title/Abstract] OR "ethylparaben" [Title/Abstract] OR "prophylparaben" [Title/Abstract] OR "buthylparaben" [Title/Abstract] OR "benzophenones" [Mesh] OR "benzophenones" [Title/Abstract] OR "benzophenone 1" [Title/Abstract] OR "2,4-dihydroxy-benzophenone" [Title/Abstract] OR "benzophenone 2" [Title/Abstract] OR "2,2',4,4'-tetrahydroxybenzophenone" [Title/Abstract] OR "benzophenone 3" [Title/Abstract] OR "oxybenzone" [Title/Abstract] OR "benzophenone 4" [Title/Abstract] OR "sulisobenzone" [Title/Abstract] OR "4-hydroxybenzophenone" [Title/Abstract] OR "benzophenone 5" [Title/Abstract] OR "benzophenone 6" [Title/Abstract] OR "2,2'-dihydroxy-4,4'-dimethoxybenzophenone" [Title/Abstract] OR "benzophenone 7" [Title/Abstract] OR "5-chloro-2-hydroxybenzophenone" [Title/Abstract] OR "benzophenone 8" [Title/Abstract] OR "dioxybenzone" [Title/Abstract] OR "benzophenone 9" [Title/Abstract] OR "benzophenone 10" [Title/Abstract] OR "benzophenone 12" [Title/Abstract] OR "octabenzone" [Title/Abstract])
#2	("Inflammation Mediators" [Mesh] OR “inflammation mediators” [Title/Abstract] OR "Inflammation" [Mesh] OR "Inflammation" [Title/Abstract] OR “cytokines” [Mesh] OR “cytokines” [Title/Abstract] OR “intracellular adhesion molecules” [Title/Abstract] OR “humoral mediators” [Title/Abstract] OR “c reactive protein” [Title/Abstract] OR “inflammatory milieu” [Title/Abstract] OR “phagocytic leukocytes” [Title/Abstract] OR “antibodies” [Title/Abstract] OR “complement proteins” [Title/Abstract] OR "receptor Activator of Nuclear Factor-kappa B" [Mesh] OR "receptor Activator of Nuclear Factor-kappa B" [Title/Abstract] OR “prostaglandin-Endoperoxide Synthases” [Mesh] OR “prostaglandin-Endoperoxide Synthases” [Title/Abstract])
**Database**	**Scopus**
**Date**	**2/1/2023**
**Strategy**	**#1 AND #2**
#1	TITLE-ABS-KEY ("bisphenols" OR "bisphenol A" OR "bisphenol S" OR "bisphenol F" OR "bisphenol A-glycidyl methacrylate" OR "bisphenol A diglycidyl ether" OR "bisphenol F diglycidyl ether" OR "benzophenones" OR "benzophenone 1" OR "2,4-dihydroxy-benzophenone" OR "benzophenone 2" OR "2,2',4,4'-tetrahydroxybenzophenone" OR "benzophenone 3" OR "oxybenzone" OR "benzophenone 4" OR "sulisobenzone" OR "4-hydroxybenzophenone" OR "benzophenone 5" OR "benzophenone 6" OR "2,2'-dihydroxy-4,4'dimethoxybenzophenone" OR "BP6" OR "benzophenone 7" OR "5-chloro-2-hydroxybenzophenone" OR "benzophenone 8" OR "dioxybenzone" OR "BP8" OR "benzophenone 9" OR "benzophenone 10" OR "benzophenone 12" OR "octabenzone" OR "parabens" OR "methylparaben" OR "ethylparaben" OR "prophylparaben" OR "buthylparaben")
#2	TITLE-ABS-KEY ("Inflammation Mediators" OR "Inflammation" OR "cytokines" OR "intracellular adhesion molecules" OR "humoral mediators" OR "c reactive protein" OR "inflammatory milieu" OR "phagocytic leukocytes" OR "antibodies" OR "complement proteins" OR "receptor Activator of Nuclear Factor-kappa B" OR "prostaglandin-Endoperoxide Synthases")
**Database**	**Web of Science**
**Date**	**2/1/2023**
**Strategy**	**(#1 OR #2) AND (#3 OR #4)**
#1	TI=("bisphenols" OR "bisphenol A" OR "bisphenol S" OR "bisphenol F" OR "bisphenol A-glycidyl methacrylate" OR "bisphenol A diglycidyl ether" OR "bisphenol F diglycidyl ether" OR "benzophenones" OR "benzophenone 1" OR "2,4-dihydroxy-benzophenone" OR "benzophenone 2" OR "2,2',4,4'-tetrahydroxybenzophenone" OR "benzophenone 3" OR "oxybenzone" OR "benzophenone 4" OR "sulisobenzone" OR "4-hydroxybenzophenone" OR "benzophenone 5" OR "benzophenone 6" OR "2,2'-dihydroxy-4,4'-dimethoxybenzophenone" OR "benzophenone 7" OR "5-chloro-2-hydroxybenzophenone" OR "benzophenone 8" OR "dioxybenzone" OR "benzophenone 9" OR "benzophenone 10" OR "benzophenone 12" OR "octabenzone" OR "parabens" OR "methylparaben" OR "ethylparaben" OR "prophylparaben" OR "buthylparaben")
	AB=("bisphenols" OR "bisphenol A" OR "bisphenol S" OR "bisphenol F" OR "bisphenol A-glycidyl methacrylate" OR "bisphenol A diglycidyl ether" OR "bisphenol F diglycidyl ether" OR "benzophenones" OR "benzophenone 1" OR "2,4-dihydroxy-benzophenone" OR "benzophenone 2" OR "2,2',4,4'-tetrahydroxybenzophenone" OR "benzophenone 3" OR "oxybenzone" OR "benzophenone 4" OR "sulisobenzone" OR "4-hydroxybenzophenone" OR "benzophenone 5" OR "benzophenone 6" OR "2,2'-dihydroxy-4,4'-dimethoxybenzophenone" OR "benzophenone 7" OR "5-chloro-2-hydroxybenzophenone" OR "benzophenone 8" OR "dioxybenzone" OR "benzophenone 9" OR "benzophenone 10" OR "benzophenone 12" OR "octabenzone" OR "parabens" OR "methylparaben" OR "ethylparaben" OR "prophylparaben" OR "buthylparaben")
#3	TI=("Inflammation Mediators" OR "Inflammation" OR “cytokines” OR “intracellular adhesion molecules” OR “humoral mediators” OR “c reactive protein” OR “inflammatory milieu” OR “ phagocytic leukocytes” OR “antibodies” OR “complement proteins” OR "receptor Activator of Nuclear Factor-kappa B" OR “ prostaglandin-Endoperoxide Synthases”)
#4	AB=("Inflammation Mediators" OR "Inflammation" OR “cytokines” OR “intracellular adhesion molecules” OR “humoral mediators” OR “c reactive protein” OR “inflammatory milieu” OR “ phagocytic leukocytes” OR “antibodies” OR “complement proteins” OR "receptor Activator of Nuclear Factor-kappa B" OR “ prostaglandin-Endoperoxide Synthases”)

Supplementary Table S2. Additional information collected from the included studies in this systematic review

Article Number	Reference	Population characteristics			Characteristics of exposure assessment			Characteristics of outcome assessment	
		Health condition	Gender	Age	Quantification methodology	Volume	LOD	Quantification methodology	FD (%)
1	Ashley-Martin et al., 2015 [56]	Healthy	Pregnant women	>30 (most of them)	GC-MS-MS	N.R.	0.20	ELISA	N.R.
2	Aung et al., 2019 [57]	Healthy	Pregnant women	>30 (most of them)	ID-LC-MS/MS	N.R.	N.R.	ELISA and Milliplex MAP High Sensitivity Human Cytokine Magnetic Bead Panel	78.0-99.9
3	Choi et al., 2017 [58]	Healthy	Men and women	30-64	LC-MS/MS	N.R.	N.R.	TIA	N.R.
4	Ferguson et al., 2016 [59]	Healthy	Pregnant women	N.R.	ID-LC-MS/MS	N.R.	N.R.	Enzyme-linked immunosorbent assay and sensitivity Human Cytokine Magnetic Bead Panel	N.R.
5	Haq et al., 2020 [60]	Healthy/diabetes	Men and women	43.86 ± 13.61*	ELISA	N.R.	N.R.	ELISA	N.R.
6	Huang et al., 2017 [61]	Healthy	Pregnant women	18-45	UPLC	N.R.	0.16	ELISA and SYNCHRON Systems reagent with the highly sensitive near infrared particle immunoassay rate methodology	0.0-100.0
7	Jain et al., 2020 [62]	Healthy/diabetes	Men and women	44.12 ± 8.5*	N.R.	N.R.	N.R.	ELISA	N.R.
8	Kelley et al., 2019 [63]	Healthy	Pregant women	18-42	LC-MS/MS	N.R.	N.R.	N.R.	N.R.
9	Lang et al., 2008 [64]	Healthy	Men and women	18-74	HPLC	N.R.	N.R.	Latexenhanced nephelometry	N.R.
10	Liang et al., 2020 [65]	URSA	Women	20-40	UPLC-MS/MS	N.R.	0.01-0.10	Multiplex assay U-PLEX proinflammatory panel 1 kit and single-plex assay V-PLEX assay.	9.0-100.0
11	Linares et al., 2021 [66]	Crohn´s disease	Men and women	40	UPLC-MS/MS	N.R.	N.R.	N.R.	N.R.
12	Mohsen et al., 2018 [67]	Healthy	Boys and girls	6-16	LC-MS/MS	500 µL	N.R.	ELISA	N.R.
13	Nalbantoğlu et al., 2021 [68]	Healthy/allergic rhinitis	Boys and girls	Healthy children: 8.32 ± 1.29*<br>Allergic rhinitis children: 8.78 ± 1.51*	ELISA	10 mL	N.R.	ELISA	N.R.
14	Qu et al., 2022 [69]	Healthy/rheumatoid arthritis	Men and women	55	UPLC-MS/MS	500 µL	0.05-0.12	Immunoturbidimetry	N.R.
15	Savastano et al., 2015 [70]	Healthy	Men	53.5 ± 5.7*	ELISA	500 µL	N.R.	Human BIO-PLEX Suspension Array Multi-panel System	N.R.
16	Šimková et al., 2020 [71]	Healthy/PCOS	Women	Healthy women : 29.9<br>PCOS women: 28.9-29.5	LC-MS/MS	500 µL	N.R.	Multiples immunoanalytic xMAP technology - kit #M500KCAF0Y Bio-Plex Pro™ Human Cytokine 27-plex Assay	N.R.
17	Song et al., 2017 [72]	Healthy	Men and women	≥60	HPLC	N.R.	0.01	High sensitive latex turbidimetric immunoassay, ELISA and autobiochemical analyser	N.R.
18	Tsen et al., 2021 [73]	Healthy	Men and women	20-45	LC-MS/MS	1 mL	0.10	Quantikine® human immunoassay kits	N.R.
19	Watkins et al., 2015 [43]	Healthy	Pregnant women	18-40	ID-LC-MS/MS	N.R.	0.20-0.40	ELISA and Milliplex MAP High Sensitivity Human Cytokine Magnetic Bead Panel	64.0-100.0
20	Yang et al., 2009 [74]	Healthy	Men and women	45.9-57.0	LC-MS/MS	500 µL	0.06	High sensitive assay	100.0

*URSA: unexplained recurrent spontaneous abortion; PCOS: Polycystic Ovary Syndrome; GC-MS-MS: gas chromatography-mass spectrometry; ID-LC-MS/MS: isotope dilution-liquid chromatography-tandem mass spectrometry; LC-MS/MS: liquid chromatography-mass spectrometry; UPLC: acquity ultraperformance liquid chromatography; UPLC-MS/MS: ultra-performance liquid chromatography-tandem mass spectrometry; ELISA: enzyme-linked immunosorbent assay; HPLC: high performance liquid chromatography; TIA: turbido-immunometric assay; LOD: limit of detection; FD: frequency of detection; N.R.: Not reported. *Mean ± standard deviation.*
